# Serum miR-128-2 Serves as a Prognostic Marker for Patients with Hepatocellular Carcinoma

**DOI:** 10.1371/journal.pone.0117274

**Published:** 2015-02-02

**Authors:** Liping Zhuang, Litao Xu, Peng Wang, Zhiqiang Meng

**Affiliations:** Department of Integrative Medicine, Fudan University Shanghai Cancer Center, Department of Oncology, Shanghai Medical College, Fudan University, Shanghai, 200032, China; University of Barcelona, SPAIN

## Abstract

Circulating miRNAs are promising biomarkers for predicting the aggressiveness of hepatocellular carcinoma (HCC). We aimed to identify differentially expressed miRNAs in the serum of HCC patients with different Barcelona Clinic Liver Cancer (BCLC) stage, and to investigate the potential of serum miRNAs as biomarkers for patient outcomes. In the discovery stage, TaqMan Low-Density Array was used to test the difference in levels of serum miRNAs between 20 patients with portal vein tumor thrombosis (PVTT) and 20 patients without PVTT. The detected serum miRNAs then were validated in 182 patients. Fifteen serum miRNAs showed more than two-fold higher expression in patients with PVTT, and miR-128-2 was found to be significantly up-regulated and was selected for further validation. In the validation stage, patients were divided into two groups with low or high serum miR-128-2 using the median expression level of all 182 cases as the cut-off point. Kaplan-Meier analysis revealed that patients with low level of serum miR-128-2 had favorable trends of survival (log rank = 13.031, p < 0.001). The median survivals for patients with a low and high level of serum miR-128-2 were 625 (95% CI, 527–722) days and 426 (95% CI, 362–491) days, respectively. MiR-128-2 was also an independent factor of overall survival (p = 0.001, HR 2.793, 95%CI 1.550, 5.033). Serum levels of the ubiquitously expressed miR-128-2 showed no significant correlation with parameters of liver damage or liver function. In addition, expressions of miR-128-2 in HCC tissues were up-regulated in comparison with adjacent non-tumor tissues. In conclusion, serum level of miR-128-2 serves as a noninvasive biomarker for the overall survival of patients with hepatocellular carcinoma.

## Introduction

Hepatocellular carcinoma (HCC) accounts for 85–90% of all primary liver cancers, is an extremely poor prognostic cancer [1, 2]. About 80% of patients present with locally advanced or metastatic disease [3]. It is mainly due to the highly vascular nature of HCC tumors, which show the propensity to spread and invade into neighboring or distant tissues [4]. In addition, underlying chronic liver disease increases the complexity and heterogeneity of HCC pathology [5]. The treatment of HCC is usually performed according to the recommendation of the Barcelona Clinic Liver Cancer (BCLC) staging system, which not only takes the tumor associated parameters into consideration, but also includes liver function and performance status. It has also been confirmed as a prognostic relevance for the overall survival in HCC patients [6, 7].

MicroRNAs (miRNAs), about 22 nucleotides long, are non-coding RNAs that negatively regulate gene expression at the post-transcriptional level [8]. Because a single miRNA can regulate hundreds of downstream genes with different biologic entities, the information gained from miRNA profiling may provide more accurate classification of cancer subtypes than the use of expression profiles of protein-coding genes [9]. In liver cancer, numerous studies have reported the association of miRNA expression profiles with the onset and progression of tumor. MiRNAs such as miR-17-5p, miR-21, miR-181b, miR-143, miR-221 and miR-224, which are associated with cell proliferation, apoptosis inhibition, and migration promotion, are found to be upregulated in HCC tissues [10–15]. Given the difficult access of tumor tissues from advanced HCC patients, circulating miRNAs are preferable noninvasive biomarkers for diagnosis and prognosis assessment. Recent findings demonstrated that human serum and plasma contain a large amount of stable miRNAs, and that the expression profile of these miRNAs holds great promise as a novel non-invasive biomarker [16]. The profile of circulating microRNAs has been explored in a variety of studies aiming to improve the early detection of HCC and to predict the response to therapy [17–19].

Venous metastasis, with tumor thrombi in the portal vein and the inferior vena cava, is a major hallmark of metastatic HCC [20]. Chen SQ et al reported that 40%–90.2% of advanced HCC patients had portal vein tumor thrombosis (PVTT) [21]. Even in patients with HCC tumor smaller than 2cm, 40.5% of them had microscopic venous invasion [22]. The presence of PVTT represents limited benefit of various treatment and poor survival outcome in HCC patients. Till now, no biomarkers related to PVTT have been reported. In this study, we hypothesize that there is a serum miRNA profile that can be used as a fingerprint for patients with different status of PVTT and survival outcomes. To address this hypothesis, we screened serum miRNAs by using TaqMan Low-Density Array (TLDA) in patients with PVTT or not, followed by an extensively validated study in a cohort of 182 patients.

## Materials and Methods

### Study subjects

HCC patients who were treated between January 2012 and July 2013 in Integrative Department, Fudan University Shanghai Cancer Center, were retrospectively enrolled into the present study. Inclusion criteria were as follows: histologically confirmed HCC or clinical diagnosis based on dynamic imaging and an underlying chronic liver disease; a good performance status (ECOG level <2); favorable liver functions of Child-Pugh class A or B. Patients with a history of another malignant within the last five years were excluded. Five milliliters of venous blood was collected from each participant at his/her first admission to the hospital. The whole blood was centrifuged at 4°C, 3000 r.p.m. for 10 min, which was followed by an additional centrifugation at 12,000 r.p.m. for 15min to completely remove all remaining cells. The serum samples were portioned in aliquots and stored at -80°C until analysis. This study was approved by the Ethics Committee of Fudan University Shanghai Cancer Center, Shanghai, China, and written informed consent was obtained from each participant, in accordance with the institutional guidelines of our hospital.

### RNA isolation

Total RNA was extracted from 500ul of serum using a miR-PARIS kit (AM1556) according to the manufacturer’s instructions for enrichment procedure for small RNAs. To allow for normalization of sample-to-sample variation in RNA isolation, synthetic Caenorhabditis elegans miRNAcel-miR-54(purchased as a custom RNA oligo nucleotide from Qiagen) was added (50pmol/l in a 5-ul total volume) to each denatured sample.

### MiRNA profiling using the TaqMan Low-Density Array

MiRNA profiling assays of two samples (100ul serum sample from each patient and then mixed together) were performed using the TLDA (Applied Biosystems, CA, USA). Each sample was analyzed with an A & B card for duplicate detection of a total of 754 miRNAs together with endogenous and negative controls. In order to increase the sensitivity of the TLDA, a pre-amplification was performed after the Megaplex reverse transcription (RT) reactions. All procedures were carried out according to the protocols recommended by the manufacturer. qRT-PCR was carried out on an Applied Biosystems 7900HT thermocycler using the recommended cycling conditions. The array data were analyzed using ∆∆CT method, DataAssist Software (Applied Biosystems).

### Quantitative real-time reverse-transcription (RT)-PCR assays

We used TaqMan miRNA probes (Applied Biosystems) to perform qRT–PCR assays according to the manufacturer’s instructions. In brief, 2 ul aliquot of enriched small RNAs from serum samples were reverse transcribed using the Taq-Man MicroRNA Reverse Transcription Kit (Applied Biosystems, San Diego, CA). Then 2 ul of the cDNA solution was used as template for the PCR stage. PCR reaction was performed using 10 ml TaqMan Universal Master Mix (2×) (Applied Biosystems, USA), 1 ul gene-specific probe, and nuclease-free H2O in a final volume of 20 ul. No-template controls for both RT step and PCR step were included to ensure target specific amplification. All reactions were run in duplicate. The CT values of the different samples were compared using the ∆∆CT method [23]. The relative expression levels of target miRNAs were normalized by cel-miR-54.

### Clinical chemistry

Standard parameters of liver function and alpha-fetoprotein (AFP) levels were measured at the central laboratory of the Fudan University Shanghai Cancer Center.

### Statistical methods

Differences in patient characteristics between two groups were evaluated by the Chi-squared test, the student’s t-test or the nonparametric Wilcoxon-Mann-Whitney test according to the variable type. We defined the follow-up duration from the date of diagnosis to the last follow up. The overall survival (OS) was calculated from the date of a definitive diagnosis to death or to the date of the last follow up. Kaplan-Meier method was used to compare the OS between patients in different groups. Only covariates significantly associated with outcomes at univariate analysis (two-sided P value<0.10) are shown and included in the multivariate model. Results were reported as hazard ratios (HR) with 95% confidence intervals (CI). Potential covariates were adjusted in multivariate model by Forward LR method.

The correlation coefficients between the expression level of miRNA and laboratory parameters were calculated by using the Spearman correlation. P values < 0.05 were considered to be significant. Data were analyzed using the SPSS version 20 (IBM, Chicago, IL).

## Results

### Detection of differentially expressed serum miRNAs in HCC patients with PVTT compared with no PVTT

TLDA was performed to identify candidate miRNAs associated with PVTT and survival in HCC patients. miRNA profiling in serum samples from 20 HCC patients with PVTT was compared with profiling in serum samples from 20 HCC patients without PVTT. In PVTT group, tumor thrombi involving the main portal vein trunk in six patients, and tumor thrombi involving right/left portal vein in another fourteen patients. Clinical variables were similar between the two groups, with the exception of the serum alpha-fetoprotein, BCLC stage and the median overall survival (Table 1). Of the 754 miRNAs incorporated in the array, 216 and 212 miRNAs were detected in serum of HCC patients with PVTT and without PVTT, respectively. Overall, 15 miRNAs showed more than 2-fold higher expression in PVTT group than no-PVTT group, and miR-128-2 was found to be significantly upregulated. Furthermore, miR-128-2 has never been reported in HCC, and its expression level in serum is detachable. Thus, it was selected for further validation (Table 2).

**Table 1:** Patients’ characteristics.

Parameter	Cohort 1(n = 40)			Cohort 2(n = 182)
	PVTT(n = 20)	No-PVTT(n = 20)	P value	
Gender, male/female	18/2	17/3	0.904	155/27
Age, years, mean±SD	50.4±7.5	53.9±9.3	0.24	53.1±10.5
Hepatitis B, n (%)	17(85)	18(90)	0.697	159 (87)
Child-pugh stage (A/B/C)	18/2/0	19/1/0	0.737	163/19/0
BCLC (B/C)	0/20	20/0	0.000	98/84
Treatment				
Local therapy, n (%)	20(100)	20(100)	1	177(97.3)
Sorafenib, n (%)	1(5)	0(0)	0.317	33(18.1)
Laboratory results				
TBIL (umol/L), mean±SD	17.7±9.46	15.7±6.1	0.856	18.3±12
DBIL(umol/L), mean±SD	8.1±6.1	5.5±3.9	0.083	7.0±7.2
ALT (IU/L), mean±SD	72.6±103.2	39.8±22.5	0.338	47.5±50.4
AST (IU/L), mean±SD	65.5±38.3	50.2±31.4	0.062	61.1±64.2
γ-GGT (IU/L), mean±SD	167.9±75	149.9±188.6	0.053	174.5±198.0
LDH (IU/L), mean±SD	268.3±117.8	207.3±97.3	0.086	240.4±146.6
ALP (IU/L), mean±SD	180.5±157.4	125.6±76.5	0.104	146.3±113.6
ALB (g/L), mean±SD	38.1±4	39.8±4.9	0.057	39.2±4.6
AFP (ng/ml), mean±SD	3011±1230	1089±1556	0.000	1415±1615
Median OS (95% CI), days	289.5(242.2, 446.9)	541.5(395.2, 829.3)	0.013	525.6(466.2,585)

**Table 2:** Serum miRNAs overexpressed in HCC patients with PVTT in comparison with no PVTT.

MiRNA	CT value for PVTT	CT value for no-PVTT	Fold-change
hsa-miR-128-2	28.9625	35.244	60.8355
hsa-miR-545	29.9643	32.9926	6.3806
hsa-miR-138	32.9851	36.0033	6.3363
hsa-miR-511	30.978	33.9827	6.2772
hsa-miR-193b	25.9747	28.9215	6.0303
hsa-miR-224	27.9724	30.0886	3.3906
hsa-miR-1267	28.0004	30.1077	3.37
hsa-miR-125b	27.9226	29.9798	3.2547
hsa-miR-148b	28.8768	30.1027	3.2133
hsa-miR-375	25.9421	27.9755	3.2016
hsa-miR-642	31.9555	33.9612	3.1408
hsa-miR-374b	31.9823	33.9681	3.0977
hsa-miR-885-5p	22.9879	24.9635	3.076
hsa-miR-381	32.9906	34.9648	3.0729
hsa-miR-625	31.0133	32.9356	2.9644

We then performed individual qRT–PCR detection on another 182 samples to quantify the serum expression of miR-128-2 in HCC patients. Most of the patients in this cohort were men (85%), were long-term carriers of hepatitis B virus (HBV) (87%). All of the patients presented with locally advanced or metastatic HCC, and preserved liver function of Child A/B. 31.3% (57/182) of patients had PVTT (Table 1). In 107 patients the diagnosis of HCC was confirmed by histopathological examination of biopsy material, whereas in another 75 patients HCC was clinically diagnosed. The mean duration of follow-up was 656±393 days.

### Serum miRNA-128-2 is a prognostic marker for HCC patients

**Fig 1:**
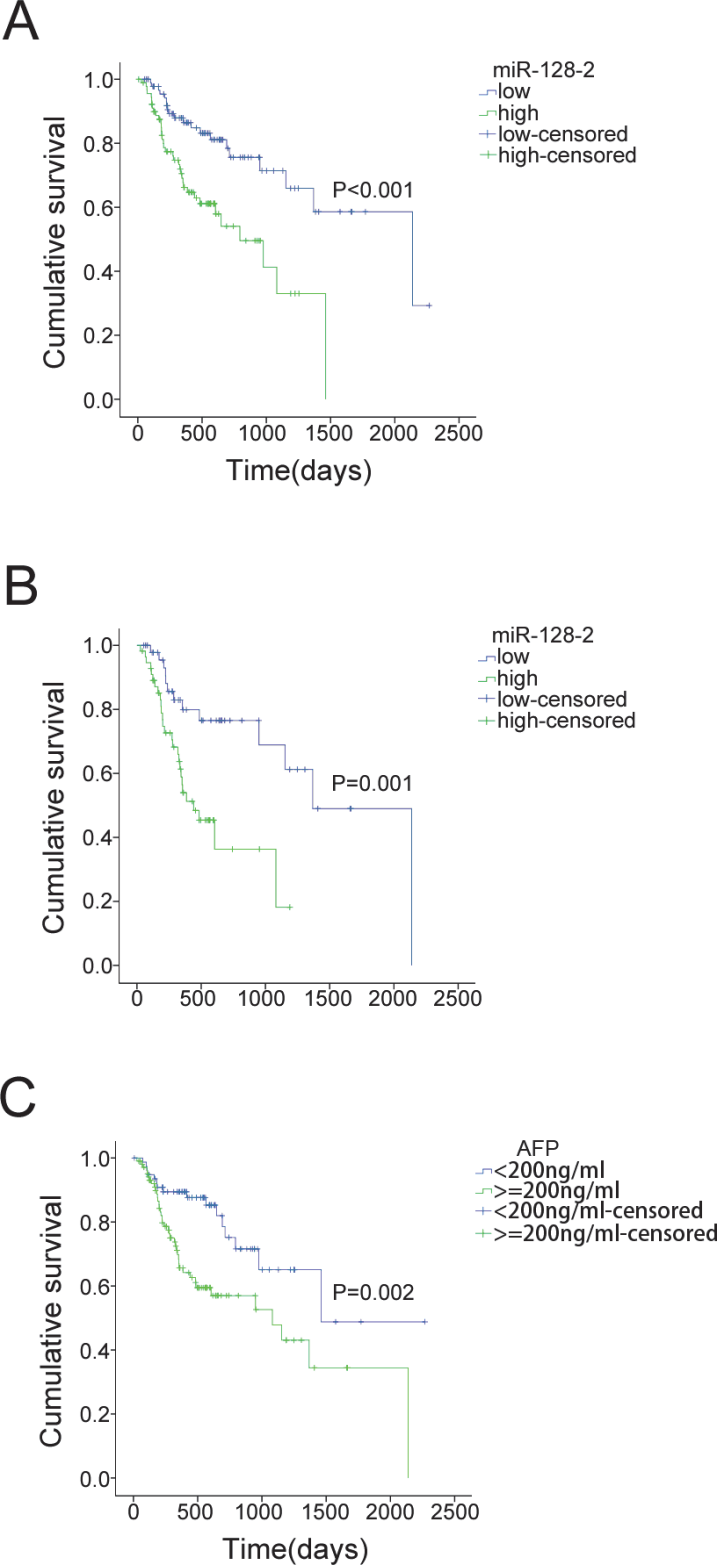
Kaplane Meier curves for different subsets of patients. **A**. Kaplane Meier curve for overall survival in a cohort of 182 HCC patients grouped by the serum level of miR-128-2. The serum miR-128-2 level was analyzed by TaqMan real-time qPCR, and the median miR-128-2 expression level was chosen as the cut-off point for separating the miR-128-2 low-level cases (n = 91) from the miR-128-2 high-level cases (n = 91). Low level of serum miR-128-2 was associated with better survival. B. Low level of serum miR-128-2 was still related with better survival in the subset of patients with serum level of AFP higher than 200ng/ml. C. Patients with serum level of AFP lower than 200ng/ml present better survival in comparison with patients with AFP level higher than 200ng/ml.

Patients were divided into two groups with low or high serum miR-128-2 using the median expression level of all 182 cases as the cut-off point. Kaplan-Meier analysis revealed that patients with low level of serum miR-128-2 had favorable trends of OS (log rank = 13.031, p < 0.001; Fig. 1A). The median survivals for patients with a low level of serum miR-128-2 and that with a high level of serum miR-128-2 were 625 (95% CI, 527–722) days and 426 (95% CI, 362–491) days, respectively. BCLC stage is a strong significant factor for HCC prognosis. We then grouped patients according to the BCLC stage and the status of PVTT, and compared the survival curves according to miR-128-2 levels in each subgroup of patients. The significant differences were all evident, which indicated that low level of serum miR-128-2 presented better survival (S1 and S2 Figs.).

To examine whether the serum miR-128-2 level was associated with specific stages of the disease, patients were classified according to the BCLC stage. No significant difference was found on the expression level of miR-128-2 between patients with stage B and stage C (p = 0.274). Similarly, the miR-128-2 level in patients with PVTT was not significantly different from HCC patients without PVTT (p = 0.733), which suggested that miR-128-2 was independent from BCLC stage. In addition, it might not associate with the presence of PVTT.

Serum AFP is a biomarker conventionally used in the diagnosis of HCC and the prediction of treatment response and survival. Following the AASLD Guidelines, an AFP level >200 ng/ml is favorable for the diagnosis of HCC when liver lesions are >2cm in size [24]. Thus, we examined the effect of miR-128-2 in two subsets of patients, using 200ng/ml of the serum AFP level as the cut-off point. In group one, which patients with serum AFP level lower than 200ng/ml, Kaplan-Meier Analysis revealed that patients with low or high level of miR-128-2 showed no significant difference in OS (log rank = 1.991, p = 0.158). Contrarily, in group two, which patients with serum AFP level higher than 200ng/ml, lower miR-128-2 presented with better survival (log rank = 10.156, p = 0.001; Fig. 1B), which indicated that the predictive effect of miR-128-2 might be dependent on serum AFP level. Further analysis found a weak positive correlation between serum miR-128-2 level and AFP levels (r = 0.198, p = 0.009, Table 3).

**Table 3:** Correlation of serum miR-128-2 levels and laboratory parameters.

Variables	miR-128-2	
		Rank correlation coefficient (r)	P-value
TBIL (umol/L)	-0.031	0.688
D-TBIL (umol/L)	0.013	0.865
ALT(IU/L)	0.144	0.059
AST(IU/L)	0.035	0.641
γ-GGT (IU/L)	0.093	0.224
LDH(IU/L)	0.125	0.101
ALP(IU/L)	0.049	0.519
ALB (g/L)	0.014	0.859
AFP(ng/ml)	0.198	0.009
PT	-0.46	0.550
INR	-0.040	0.601

TBIL, total bilirubin

D-TBIL, direct bilirubin

ALT, alanine aminotransferase

AST, aspartate aminotransferase

γ-GGT, γ-glutamyl transferase

LDH, lactic dehydrogenase

ALP, alkaline phosphatase

ALB, albumin

PT, prothrombintime activity percentage

INR, international normalized ratio.

Because serum AFP level affects prognosis (log rank = 9.214, p = 0.002; Fig. 1C) and to exclude confounding effects, we then performed a Cox proportional hazards regression analysis. A multivariate analysis confirmed that the serum miR-128-2 level was an independent prognostic factor for OS (p = 0.001, HR 2.793, 95%CI 1.550, 5.033). In addition, multivariate analysis further confirmed that low BCLC stage and local therapy were independent factors for better survival of HCC patients (Table 4).

**Table 4:** Univariate and multivariate Cox regression analyses of parameters associated with overall survival of all HCC patients.

Parameters	Univariate analysis		Multivariate analysis	
	HR(95% CI)	p-value	HR(95% CI)	p-value
Gender (male vs. female)	1.659(0.66, 4.172)	0.282		
HBV (no vs. yes)	0.767(0.375, 1.567)	0.466		
Cirrhosis (no vs. yes)	1.268(0.506, 3.181)	0.613		
AFP (<200 vs. >200ng/ml)	2.397(1.339, 4.289)	0.003	1.757(0.940, 3.284)	0.077
Child-pugh (A vs. B)	2.04(1.041, 3.996)	0.038	1.500(0.758, 2.969)	0.244
PVTT (no vs. yes)	3.145(1.85, 5.345)	0.000	1.475(0.776, 2.802)	0.236
BCLC (B vs. C)	4.083(2.228, 7.482)	0.000	2.481(1.166, 5.280)	0.018
Local therapy	0.235(0.084, 0.654)	0.006	0.227(0.075, 0.687)	0.009
Sorafenib	1.423(0.791, 2.562)	0.239		
Serum miR-128-2 (low vs. high)	2.791(1.577, 4.941)	0.000	2.793(1.550, 5.033)	0.001

### Serum level of miR-128-2 didn’t correlate with inflammatory activity in the liver and its function

To investigate if the prognostic significance of serum miR-128-2 correlated with inflammatory activity in the liver and its function, we assessed parameters of total bilirubin (TBIL), direct bilirubin (D-BIL), alanine aminotransferase (ALT), aspartate aminotransferase (AST), γ-glutamyl transferase (γ-GGT), lactic dehydrogenase (LDH), alkaline phosphatase (ALP), albumin (ALB), prothrombintime activity percentage (PT), and international normalized ratio (INR) (Table 3). Serum levels of the ubiquitously expressed miR-128-2 showed no significant correlation with parameters of liver damage or liver function.

### Expression of miR-128-2 up-regulated in HCC tissues

**Fig 2:**
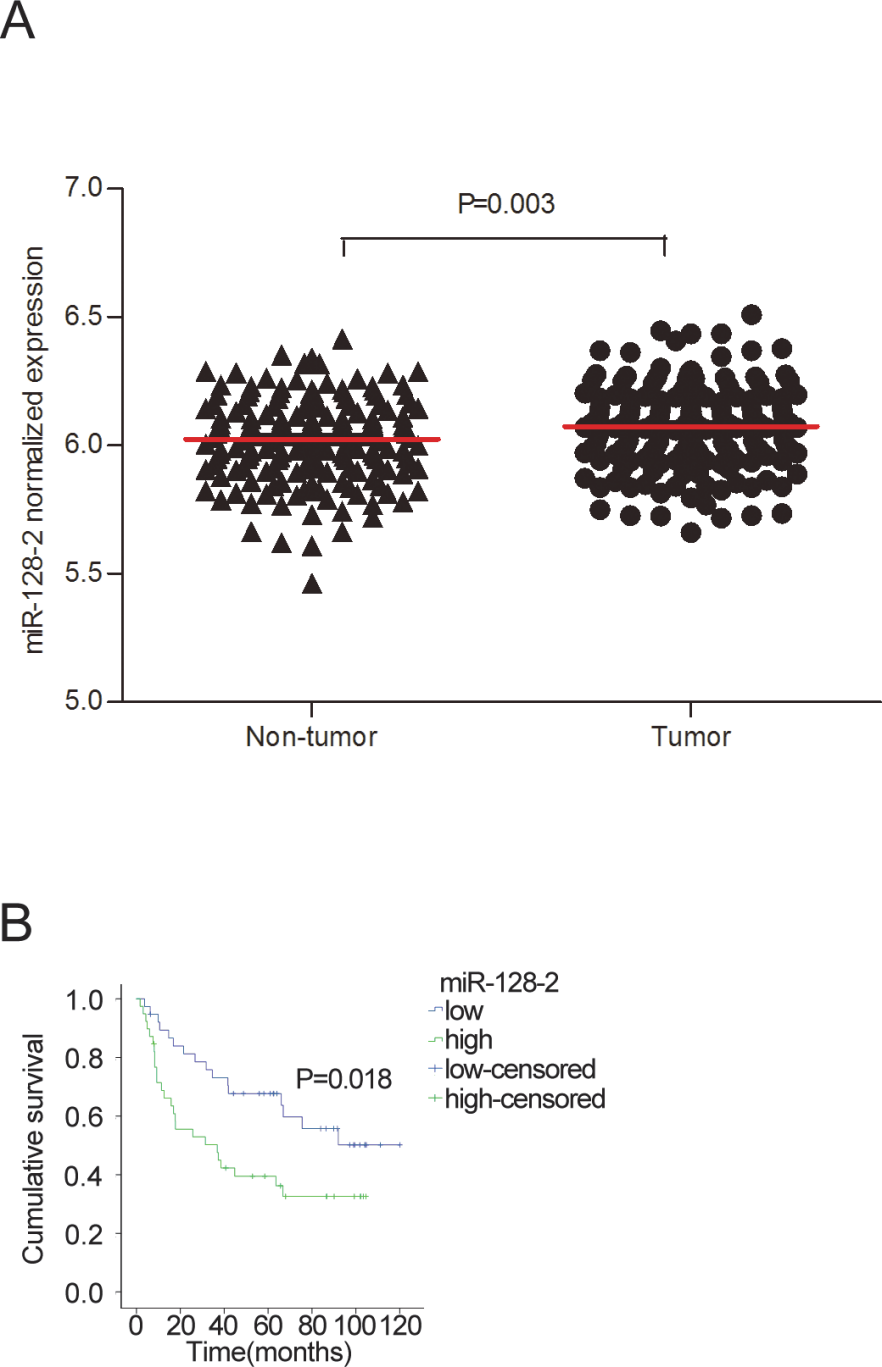
miR-128-2 expression was up-regulated in tumor tissues and correlates with shorter survival in patients with advanced HCC. **A**. A comparison of miR-128-2 expression levels in HCC tissues and adjacent non-cancer tissues. Statistical significance was calculated. B. A Kaplane Meier survival analysis of the HCC patients with BCLC B/C stage grouped according to the expression level of miR-128-2 in the tumor tissue. The median miR-128-2 expression level of the 77 cases was chosen as the cut-off point for separating the miR-128-2 low-expression tumors (n = 39) from miR-128-2 high-expression tumors (n = 38). Low level of miR-128-2 in HCC tissue was associated with better survival.

To identify the miR-128-2 expression level in HCC tissues, we analyzed the miRNA expression in a large number of human HCC samples from the GEO, a public functional genomics data repository supporting MIAME- compliant data submissions. The miRNA expression profiles of 181 tumors and the paired non-tumor liver were obtained from the study GSE36376 [25]. All data were quartile normalized. The baseline characteristics of patients were shown on S1 Table. The analysis revealed that miR-128-2was significantly overexpressed when compared with adjacent non-tumor tissue (Fig. 2A). In addition, patients were grouped in subjects with low or high tissue miR-128-2 using the median expression level of the 181 cases as the cut-off point and Kaplan-Meier analysis was done. The OS of patients with low or high tissue miR-128-2 wasn’t significantly different (P = 0.704). We then analyzed the relation between miR-128-2 expression and survival in the subgroup of patients with BCLC B or C stage. Finally, 77 cases were included in the analysis, and the median expression level of the 77 cases was used as the cut-off point. The results showed that patients with lower miR-128-2 were associated with better survival outcome (log rank = 5.594, p = 0.018; Fig. 2B). Taken together, miR-128-2 was upregualted in HCC tissues. Higher expression level of miR-128-2 is associated with a poor prognosis in patients with advanced HCC.

## Discussion

The aim of this study was to identify differentially expressed miRNAs in the serum of HCC patients with different BCLC stage, and investigate the potential of serum miRNA as a biomarker for venous metastasis and survival. We firstly conducted an array in two subsets of HCC patients. One subset of HCC patients was diagnosed at BCLC B stage, with a relatively longer survival, and the other subset was diagnosed at BCLC C stage (with PVTT but not extra liver metastasis), with a shorter survival. Thus, the differentially expressed miRNAs were prospected to be associated with tumor progression and survival outcome. The systematic screening found that miR-128-2 was the most significantly up-regulated miRNA in the serum of patients with BCLC C stage. In further validation, we confirmed that serum miR-128-2 level could be a new prognostic parameter in HCC patients that is independent from BCLC stage.

MiR-128 is encoded by two distinct genes, miR-128-1 and miR-128-2, which are processed into an identical mature sequence [26]. MiR-128 is frequently reported as a tumor suppresser in the malignance of nervous system, for example neuroblastoma, glioblastoma and medulloblastoma [27–29]. In contrast to these, Volinia S et al conducted a large-scale miRnome analysis on 540 samples including lung, breast, stomach, prostate, colon, and pancreatic tumors. They identified that elevated miR-128b was shared in colon, lung, and pancreas cancer [30]. The role of miR-128 in liver cancer has not yet been reported. We used the public data from GEO to analyze the expression level of miR-128 in HCC tissues. The results revealed that miR-128-2 was significantly overexpressed in HCC tissues in comparison with the adjacent non-tumor tissues, which indicated that miR-128 was related with the pathogenesis of HCC. However, it is still unknown whether the dysregulation of miR-128 is a cause or effect of the cancer.

The presence of PVTT is strongly correlated with a poor prognosis for HCC patients [31, 32]. Aberrantly expressed miRNAs contribute to the formation of PVTT has ever been reported. Liu S et al. comparatively analyzed the miRNA and mRNA expression profile of PVTT and the corresponding parenchyma tumor tissue. They found that miR-135a showed the greatest increase in PVTT tissue. Further in vitro and in vivo studies demonstrated that overexpression of miR-135a favored invasive and metastatic behavior [33]. PVTT cases in the literature are usually from developing countries. Yang P et al. uncovered a causative link between HBV infection and development of PVTT. Mechanistically, elevated TGF-b activity, resulting from the persistent presence of HBV in the liver tissue, suppresses the expression of microRNA-34a, leading to enhanced production of chemokine CCL22, which recruits regulatory T cells to facilitate immune escape [34]. Circulating miRNA profile in HCC patients with PVTT has not yet been reported. Initially, our study aimed to indentify certain serum miRNAs, which differentially express in patients with PVTT in comparison with no-PVTT. Then further validate whether it can be a biomarker for PVTT development and survival. Serum miR-128-2 showed the greatest increase in the screening. However, it failed to show association with the presence of PVTT in further validation. Biomarkers reflected the aggressiveness of tumor are likely to provide more accurate information for the prognosis of HCC patients. Since its stability in circulation and the easy access of blood samples, circulating miRNAs hold great promise. Circulating miRNA expression levels are not always consistent with corresponding tissue. For example, miR-122, a liver specific miRNA, has been reported as downregulated in rodent and human HCC tissues when compared with adjacent non-tumor tissues [35]. However, an elevation of serum level of miR-122 was evident in HCC patients [36, 37]. It indicates that circulating miRNAs may not always directly associate with changes occurring in tumor tissues but may also reflect indirect effects. In present study, we found a consistent expression level of miR-128-2 in serum and HCC tissues.

In conclusion, we identified serum miR-128-2 as an independent prognostic parameter for OS in HCC patients.

## Supporting Information

S1 Fig High level of serum miR-128-2 was associated with poor survival in subsets of patients grouped with BCLC stage. Patients with high level of serum miR-128-2 had poor survival in subset of patients with BCLC stage B and subset of patients with BCLC stage C.
(TIF)

S2 Fig High level of serum miR-128-2 was associated with poor survival in subsets of patients grouped with the status of PVTT. Patients with high level of serum miR-128-2 had poor survival in the subset of patients with PVTT and subset of patients without PVTT.
(TIF)

S1 Table Baseline characteristics of 181 samples from GEO. (DOC)
