# Supplementary figures and images for: Serum miR-128-2 Serves as a Prognostic Marker for Patients with Hepatocellular Carcinoma
Source: PLoS One. 2015 Feb 2;10(2):e0117274. doi: 10.1371/journal.pone.0117274 (PMC4313939; doi:10.1371/journal.pone.0117274)

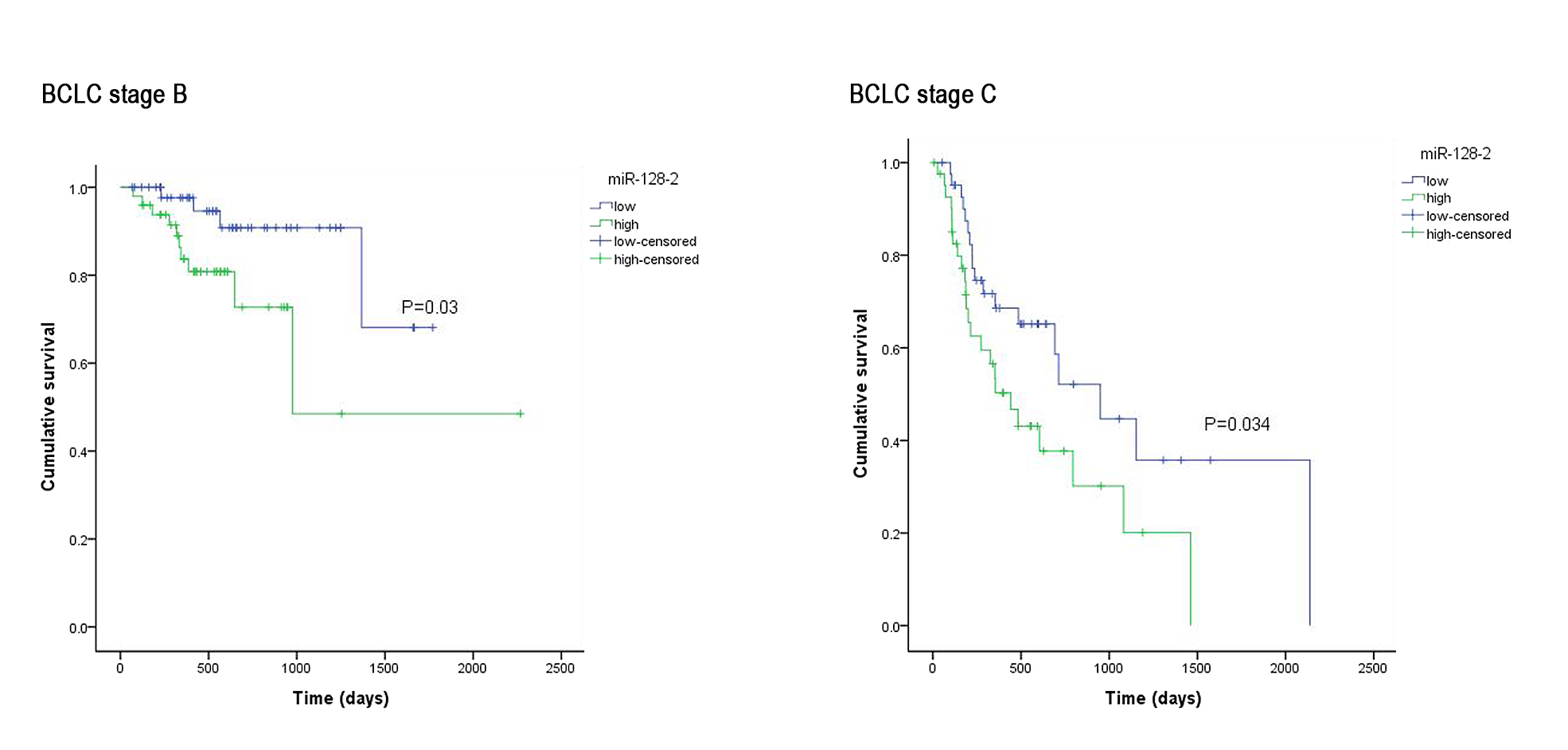

Supplement: S1 Fig — Patients with high level of serum miR-128-2 had poor survival in subset of patients with BCLC stage B and subset of patients with BCLC stage C. (TIF) [file pone.0117274.s001.tif]

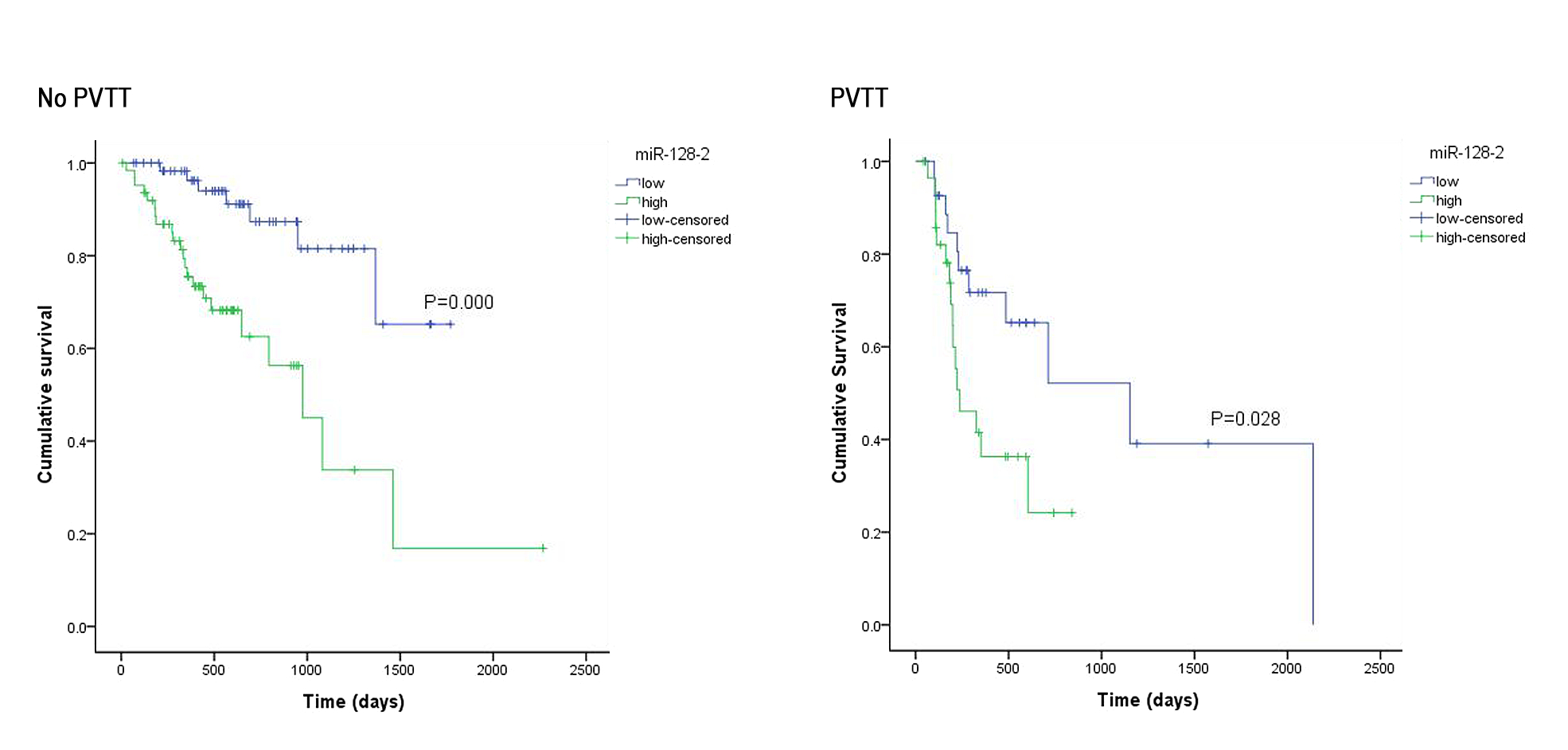

Supplement: S2 Fig — Patients with high level of serum miR-128-2 had poor survival in the subset of patients with PVTT and subset of patients without PVTT. (TIF) [file pone.0117274.s002.tif]
