# Supplementary material for: Serum miR-128-2 Serves as a Prognostic Marker for Patients with Hepatocellular Carcinoma
Source: PLoS One. 2015 Feb 2;10(2):e0117274. doi: 10.1371/journal.pone.0117274 (PMC4313939; doi:10.1371/journal.pone.0117274)
Supplement: S1 Table — (DOC) [file pone.0117274.s003.doc]

**Table S1 Baseline characteristics of 181 samples from GEO**

| **Parameter** |  |
| --- | --- |
| Gender, male/female | 151/30 |
| Age, years, mean±SD | 52.59±10.86 |
| Hepatitis B, n (%) | 146 (80.7) |
| Child-pugh stage (A/B/C) | 181/0/0 |
| BCLC (A/B/C) | 104/68/9 |
| Median OS (95% CI ),months | 68.4(63.1,73.3) |
